# Computational Models of Readers' Apperceptive Mass

**DOI:** 10.3389/frai.2022.718690

**Published:** 2022-02-22

**Authors:** Arthur M. Jacobs, Annette Kinder

**Affiliations:** ^1^Experimental and Neurocognitive Psychology Group, Department of Educational Science and Psychology, Freie Universität Berlin, Berlin, Germany; ^2^Center for Cognitive Neuroscience Berlin (CCNB), Freie Universität Berlin, Berlin, Germany; ^3^Learning Psychology Group, Department of Educational Science and Psychology, Freie Universität Berlin, Berlin, Germany

**Keywords:** distributed semantic models, apperceptive mass, childLex, digital humanities, machine learning, literary reading, SentiArt

## Abstract

Recent progress in machine-learning-based distributed semantic models (DSMs) offers new ways to simulate the *apperceptive mass* (AM; Kintsch, 1980) of reader groups or individual readers and to predict their performance in reading-related tasks. The AM integrates the mental lexicon with world knowledge, as for example, acquired *via* reading books. Following pioneering work by Denhière and Lemaire (2004), here, we computed DSMs based on a representative corpus of German children and youth literature (Jacobs et al., 2020) as null models of the part of the AM that represents distributional semantic input, for readers of different reading ages (grades 1–2, 3–4, and 5–6). After a series of DSM quality tests, we evaluated the performance of these models quantitatively in various tasks to simulate the different reader groups' hypothetical semantic and syntactic skills. In a final study, we compared the models' performance with that of human adult and children readers in two rating tasks. Overall, the results show that with increasing reading age performance in practically all tasks becomes better. The approach taken in these studies reveals the limits of DSMs for simulating human AM and their potential for applications in scientific studies of literature, research in education, or developmental science.

*The nature of the comprehension process is determined, most of all, by how well the message can be apperceived, that is, integrated into a knowledge structure*.

— Walter Kintsch, “Learning From Text, Levels Of Comprehension, Or: Why Anyone Would Read A Story Anyway,” 1980

## Introduction

Imagine a child who has read only one book, the Bible. On top of the child's biosociocultural development, this hypothetical singular reading education will have measurable consequences for the child's thoughts (e.g., concrete and abstract concepts), feelings (e.g., basic and mixed emotions), and behavior (e.g., communication). Such consequences can be assessed by various tests (e.g., active/passive vocabulary, semantic arithmetic, and analogical reasoning), and the child's performance in these tests can be predicted *via* quantitative narrative and advanced sentiment analysis of the only text it knows. This is what this paper is about: using distributed semantic (vector space) models (DSMs) trained on representative book corpora as potent *null models* of an important part of human semantic memory, or as Kintsch (1980) preferred to call it, the *apperceptive mass* (AM) of readers: this term highlights the integration of *world knowledge* as for example, acquired *via* reading books into semantic memory. This allows us to simulate and quantitatively predict the performance of individual readers or reader groups in standard linguistic or intelligence tests.

Since in general, psychological research lacks longitudinal data about a child's biosociocultural development, her communications, and so on, one can hardly estimate the experiential component of her AM. However, the AM likely results from a yet unknown integration of both experiential and distributional data, at least partially represented in associative activation patterns of semantic networks, as assumed by the semantics theory of Andrews et al. (2009). Thus, for example, the emotional valence of a word could be computed from (1) neural activation patterns distributed over the sensory-motor representations of a word's referents (*experiential* aspect) and (2) the linguistic company the words keep (Harris, 1951; Firth, 1957), that is, the size and density of their context (*distributional* aspect), as computationally modeled using cooccurrence statistics (e.g., Denhière and Lemaire, 2004; Westbury et al., 2015; Hofmann et al., 2018). This distributional component of the AM can be simulated and the performance of this null model can then serve as a benchmark for more sophisticated (process) models of the mental lexicon or AM.

**Figure 1:**
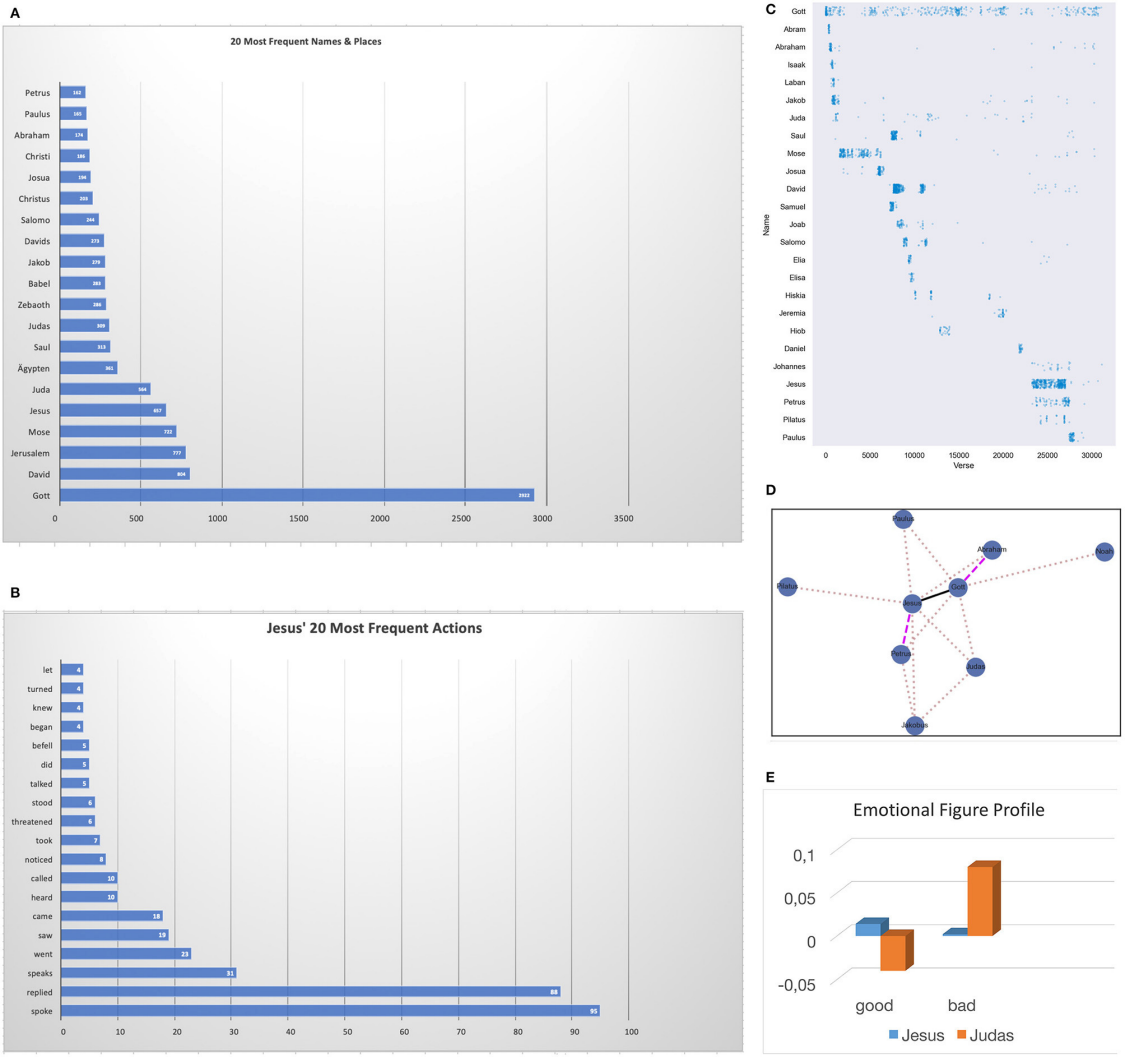
**(A)** Twenty most frequent names and places in the Bible (German Luther Bibel). **(B)** 20 most frequent actions of Jesus in the Bible. **(C)** Appearance density of 25 major characters in the Bible ordered according to verse number^a^. **(D)** Interaction network of nine major characters in the Bible (interaction frequency is represented by line width: bold >dashed>dotted). **(E)** Emotional figure profiles for Jesus and Judas computed with SentiArt (Jacobs, 2019). ^a^cf. https://pmbaumgartner.github.io/blogfholy-nlp/.

To come back to our extreme hypothetical example, if the child had read the Bible carefully and repeatedly, she could integrate lots of things into her AM, such as the names of at least the 20 most frequently mentioned persons (of ~2k) and places in the Bible (Figure 1A), or the typical actions Jesus is most often associated with (e.g., “Jesus spoke,” Figure 1B). The associative networks of her brain will also have encoded lots of other pieces of information that can constitute more or less implicit knowledge about when major characters appeared in the Bible (Figure 1C; ordered according to verse number), or with whom they typically interacted (Figure 1D). Thus, the child could remember that the most popular action in the entire Bible is *speaking—*including Jesus—but Jesus also may be remembered to have *threatened* a few times. Also, he would likely remember that Jesus was closer to Petrus than to Paulus, or that Abraham was closer to God (Gott) than Noah (Figure 1D). Finally, she may also have an opinion on the main protagonists' emotional figure profiles (cf. Jacobs, 2019), for example, whether Jesus and Judas were “good” or “bad” persons (Figure 1E).

Now, what if by chance this child got her hands on say the books of the Harry Potter series (e.g., Rowling, 1997) and wanted to read them. With a limited vocabulary of only ~27k (unique) words, she would fully understand just about 10% of the ~85k words included in the series. As unrealistic this example may appear, imagine we had reliable data about the reading materials of a person for the last 10 years (newspapers, websites, books, etc.), and then, we could construct reader-specific DSMs and predict individual reading behavior with remarkable accuracy (Hofmann et al., 2020)—but also, of course—make sophisticated guesses about this person's opinions, preferences, etc., in other words things that big internet companies already use to further their business.

Of course, the above “Bible child” example is academic, although the estimate of a passive vocabulary of ~27k may be an overstatement depending on the child's age and education. According to empirical studies summarized in Denhière et al. (2007; cf. De La Haye, 2003), an average child reading about 20 min per day learns about 900 root words (i.e., unique words) per year leading to a “normal” vocabulary of maximally 15k root words (63% nouns, 17% verbs, and 20% adjectives and adverbs) at the level of 12th grade (2nd grade: ~5k, 5th grade: ~8k). A more recent study estimated the average vocabulary size of a 20-year-old native speaker of American English as being ~40k words (Brysbaert et al., 2016). Thus, our hypothetical Bible child's vocabulary would range between that of an average 12th grader and a 20-year-old adult as far as sheer size is concerned, but the contents matter, too, of course. Thus, roughly 10% of the Bible child's vocabulary are reserved for names and only about 1/3 are nouns (without names), 1/3 are verbs, and 18% are adjectives and adverbs. Having given an idea of what quantitative narrative and advanced sentiment analysis can be used for when analyzing hypothetical individual readers, next we propose ways to examine the quality of more general DSMs, a necessary condition for using them as predictive models of reader group behavior.

## Book Corpora as Reader Group Models

The developmental lexicon project (Schröter and Schroeder, 2017) aims at helping researchers to advance theories and computational models of visual word recognition and the mental lexicon that include a developmental perspective. This paper contributes to this perspective by providing data about the performance of reader group *null models* based on a specific book corpus, that is, minimalistic and arguably unrealistic models that provide a benchmark for more sophisticated competitors. Additionally, they could also serve as *normative models*, for example, for educational purposes, for example, regarding their performance in the analogical reasoning tests of Study 3 below.

In contrast to the English children and youth literature subcorpus of the Gutenberg Literary English Corpus (GLEC; Jacobs, 2018a; Jacobs et al., 2020), the 500 books in the German childLex corpus (CL in the following; Schroeder et al., 2015) which underlies the present models mainly contain postwar and contemporary exemplars such as the seven books from the Harry Potter series (e.g., Rowling, 1997). They also include a nice mix of texts by a large variety of well-known and less well-known German and translated international writers (*N* = 248) such as Alexandre Dumas, Kirsten Boie, Erich Kästner, Ottfried Preussler, Enid Blyton, or Antoine de Saint-Exupéry. The 500 CL books vary widely in terms of length and content. A typical book for beginning readers (reading age 6–8, henceforth RA1, grades 1–2) would contain around 5k words; a book for intermediate readers ~15k words (reading age 9–10, henceforth RA2, grades 3–4); and a book for experienced readers ~50k words (reading age 11–12, henceforth RA3, grades 5–6). To ensure a sufficient number of words in each age group, CL books for the beginning and intermediate readers were oversampled, that is, contained a larger number of books (RA1: 44%, RA2: 41%, and RA3: 15%, respectively).

The texts in all three subcorpora (the RA3 model included both the RA1 and RA2 corpora just as RA2 included RA1) were preprocessed applying standard python NLP tools, for example, words were POS-tagged using *treetagger* (Schmid, 1995) and then used for DSM training. Whereas, Denhière and Lemaire (2004) in their pioneering work used a model based on latent semantic analysis (Landauer and Dumais, 1997), here we used the more recent *gensim* library with the default parameter set (Rehurek and Sojka, 2010) to generate 300d *word2vec* skipgram DSMs (Mikolov et al., 2013) which we already applied successfully in previous studies (e.g., Jacobs, 2017, 2018b, 2019; Hofmann et al., 2018; Jacobs and Kinder, 2019)^1^. Naturally, the three corpora differ in a number of lexico-semantic or syntactic features that influence the readability of the books (cf. Schroeder et al., 2015). As a control against which to gauge the performance of our CL models, we used the *sdewac* model with ~1.5 million types trained on ~45 million sentences from unspecified German texts from the web (Baroni et al., 2009). Some basic features of the corpora and models are listed in Table 1.

**Table 1:** Basic features of the corpora, the three CL models, and the control model.

**Model/test**	**SDEWAC**	**CL reading age 1**	**CL reading age 2**	**CL reading age 3**
Books	–	221	423	500
Sentences	45 million	89.012	314.596	648.613
Tokens, types	~1.5 billion, ~1.5 million	1.217.002, 55.222	4.614.548, 131.735	10.162.991, 195.662
DSM vocabulary size	1.592.753	50.430	100.338	158.615

## Study 1. Estimates of Semantic Complexity

Size (i.e., number of books) matters, but content, variety, genre, or style are equally important for a readers' education and AM, for example, regarding their ability to deal with figurative language (Kintsch, 2008; Jacobs and Kinder, 2017, 2018a). One feature of books that reflects such aspects of literary quality is semantic complexity that can be estimated *via* different measures such as *intertextual variance* or *stepwise distance* (van Cranenburgh et al., 2019). To compute the average semantic complexity score for our three corpora, we followed van Cranenburgh et al. (2019) in choosing stepwise distance as the crucial measure which turned out to be the best predictor of human *literariness ratings* for hundreds of Dutch novels.^2^ The idea was that this measure can estimate a reader group's increasing ability (from RA1 to RA3) to deal with semantic complexity and to perform various semantic tasks used in intelligence or language tests, such as analogical reasoning (see Study 3).

**Figure 2:**
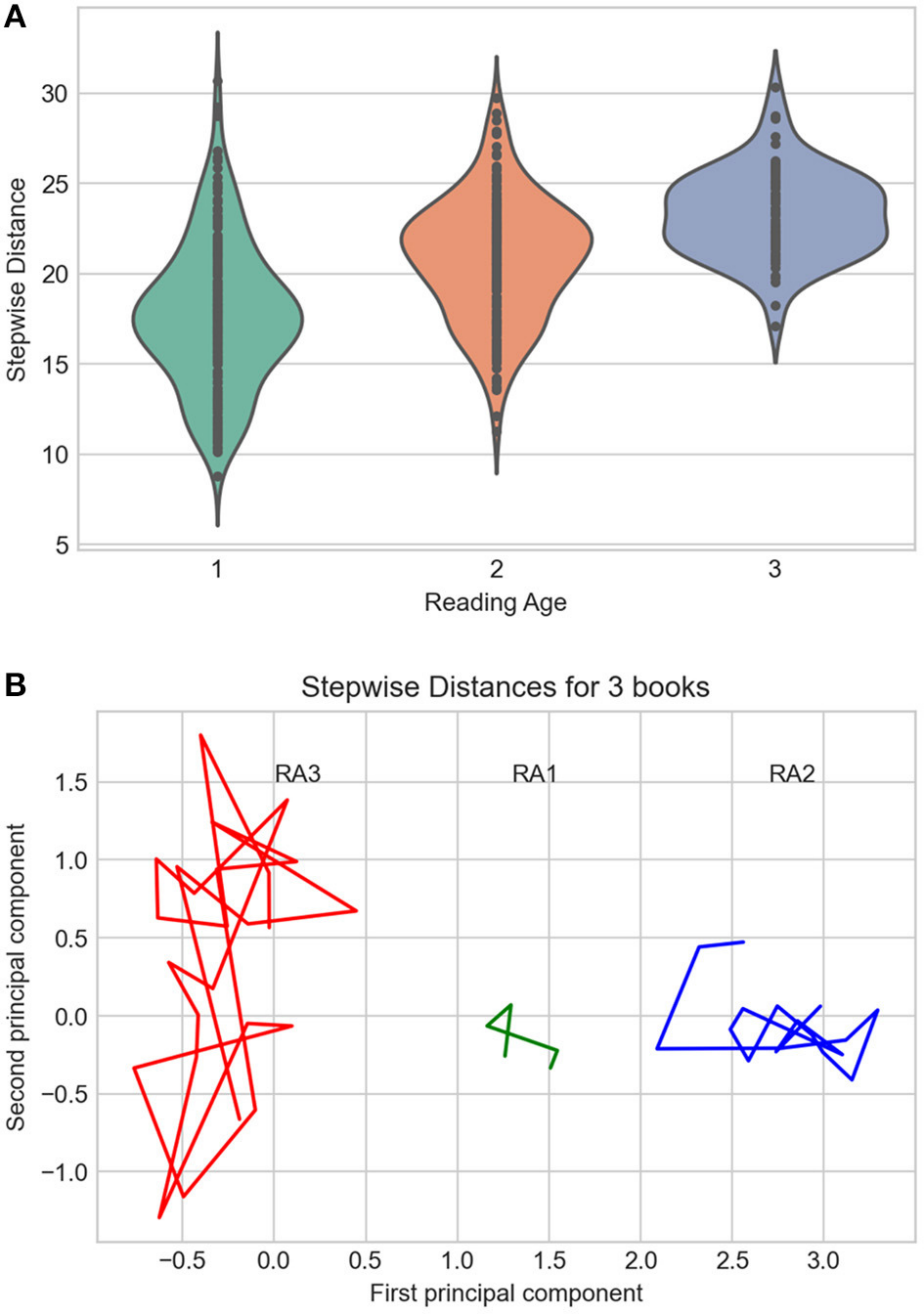
**(A)** Violin plot of stepwise distances for the three CL corpora. **(B)** Principal Component Analysis (two first components only) of stepwise distances for three representative books from each CL subcorpus. Distances between points represent semantic variance, the focus being on distances between consecutive text chunks.

The overall scores for our three RA groups were as follows: RA1 = 17.7, RA2 = 20.8, RA3 = 23.3 (all statistical differences significant at *p* < 0.0001; see Figure 2A), which suggests an increasing semantic complexity, as could be expected. According to the scores shown in Figure 2A, our RA3 model thus should perform best in semantic tests, followed by RA2 and RA1. For illustrative purposes, Figure 2B displays representative examples for three books showing how stepwise distance (shown here for the first 5–25 consecutive chunks) can differ between the three RA groups (RA3 = 27.6. Margot_Berger: Letzte_Chance_für_Jana; RA2 = 11.2. Kirsten_Boie: Jannis_und_der_ziemlich_kleine_Einbrecher, RA1 = 8.7. Bettina_Obrecht: Zwei_Freunde_für_Anna). The book representative of RA3 clearly shows a greater semantic complexity than the books standing for RA2 and RA1 on this measure of average stepwise distance.

To summarize Study 1, using a recently empirically validated global measure of the semantic complexity of books (as one proxy for literariness; van Cranenburgh et al., 2019), we showed that together with other global measures such as the number of sentences or types, it increases with reading age. This supports our assumption that the RA3 model should perform best in the semantic tests applied in Study 3.

## Study 2. Concrete, Abstract, and Emotion Concepts

Distributed semantic models can differ on a number of features or hyperparameters. It is therefore important to check their quality before applying them as models of cognitive representations. Following the approach of an earlier study (Jacobs and Kinder, 2019), here we use both a first qualitative and a subsequent quantitative evaluation (Study 3) of our DSMs. As a first simple and intuitive check, we looked at how well the DSMs distinguish between some exemplary concrete, abstract, and emotion concepts using dimension reduction techniques (TSNE; Van der Maaten and Hinton, 2008)^3^. In Study 2, we renounced on extensive quantitative tests using concept categorization benchmarks such as the Battig test (Baroni and Lenci, 2010) because they do not explicitly differentiate between concrete, abstract, and emotion concepts we were interested in. Given that we only looked at a few exemplary cases that have no single gold category, we also did not apply quantitative measures such as purity, that is, the extent to which a cluster contains concepts from a single gold category. It should be noted that more than 150 years after Darwin's emotion theory proposing the six basic emotion categories used in Figures 3, 4, psychological research still debates whether there are six, eight, or 12 basic emotions and how these could precisely be distinguished from non-basic emotions. Moreover, as far as we know, there is a single published study presenting norms for the categorization of discrete emotion terms in German (Briesemeister et al., 2011), but that is no basis for establishing international “gold standards.”

**Figure 3:**
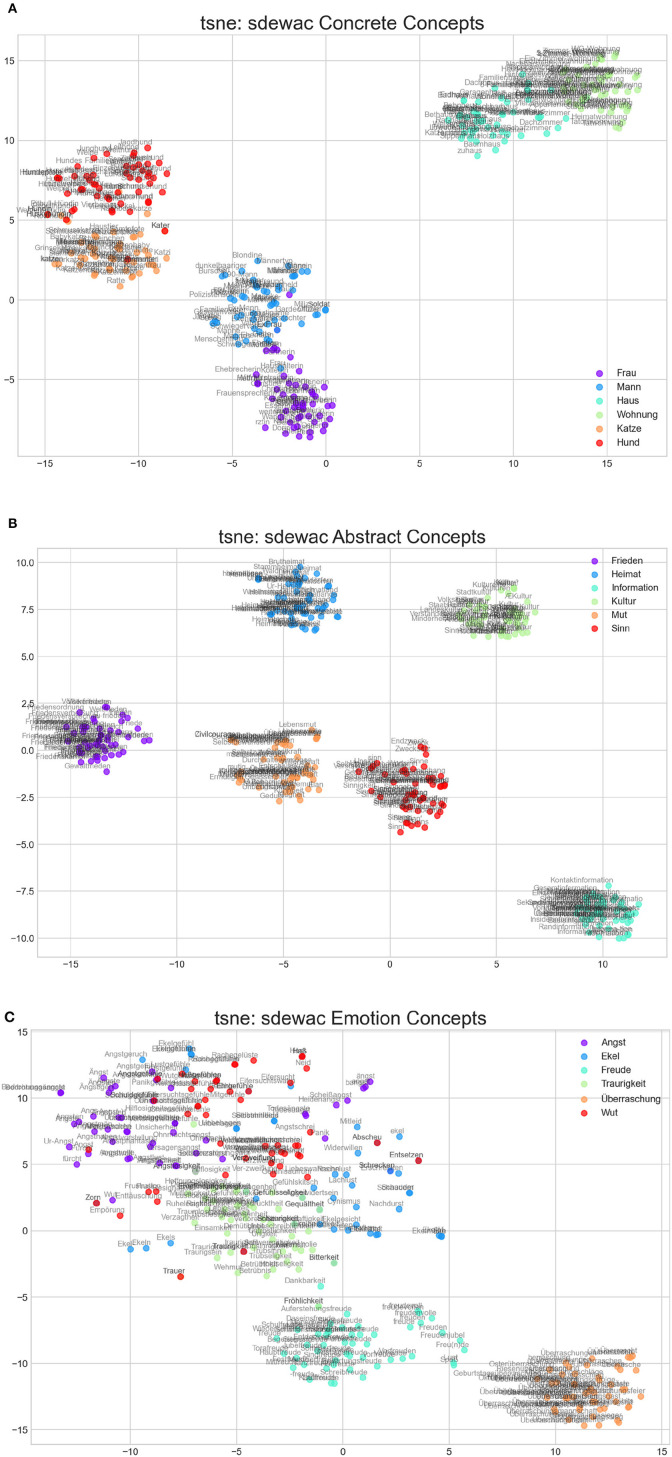
tsne representations of the semantic space of the sdewac model for exemplary concrete **(A)**, abstract **(B)**, and emotion concepts **(C)**. The words that are most similar to the target words (e. g., to woman/Frau or to man/Mann) are plotted inthe same color.

**Figure 4:**
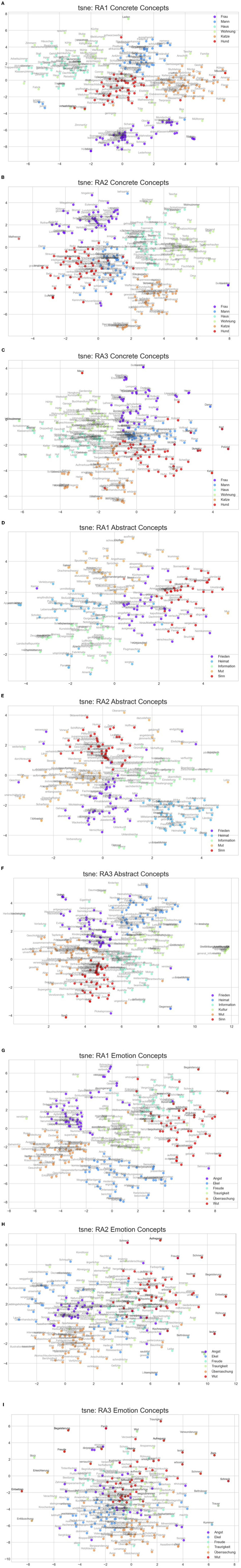
**(A–I)** tsne representations of the semantic space of the three CL models for exemplary concrete, abstract, and emotion concepts.

Take the example of the term “Mitleid” (pity). Our control DSM puts it in Darwin's “Ekel” (disgust) category (see Figure 3C). The data from Briesemeister et al. (2011) show the following ratings (scale of 1–5) for MITLEID: joy (1.5), anger (1.47), sadness (2.5), fear (1.5), and disgust (1.1). Thus, according to these data, the DSM's choice would be a miscategorization. However, the fact that the highest rating for pity is only 2.5/5 (sadness) suggests that humans have a lot of uncertainty regarding the “true” or “gold” category of that emotion term. Thus, so far neither psychological nor neuroscientific research provides something like gold standards for emotion terms, but computational data like those in Figures 3, 4 can be used for predicting those of future empirical studies on verbal emotion category learning in children and adults.

As a control against which to gauge the performance of our CL DSMs, we used the *sdewac* model (Baroni et al., 2009) which had performed best among three DSMs on a series of semantic and predictive modeling tests (Jacobs and Kinder, 2019). Figures 3, 4 summarize the main results of these exemplary qualitative analyses. As illustrated in Figure 3, the control DSM offers clear concrete (e.g., linearly separable animate vs. inanimate and human vs. animal clusters) and very distinct abstract concepts. As could be expected from our previous study though, the rather abstract emotion concepts show more overlap. Theoretically, this increased overlap is interesting and can mean at least two things: first, it could be due to emotion concepts being generally fuzzier than other concrete or abstract concepts. Second, it could mean that emotion concepts are more dynamic, flexible, or differentiated than others perhaps fulfilling an adaptive function. Future work along the lines of Briesemeister et al. (2011), Nook et al. (2017), Huebner and Willits (2018), or Hoemann et al. (2020) is necessary to decide between these options.

Interestingly for purposes of sentiment analyses, our control DSM clusters the only two positive emotions among our set (joy/Freude: cyan dots and surprise/Überraschung: orange dots) clearly apart from the four negative emotions. Regarding the latter, sadness/Traurigkeit (green) is pretty well-clustered, whereas disgust/Ekel (blue), anger/Wut (red), and fear/Angst (magenta) are more distributed.

Regarding the three CL models, the picture is more complex (Figure 4). The RA1 model shows an approximately (non-perfect) linear separability between the two animate concept groups (humans and animals) and the inanimate group (house, apartment). The model also distinguishes between the six abstract concepts, albeit in a fuzzier or more differentiated way than for the concrete ones—as could be expected. Due to vocabulary limitations, it still lacks a concept for culture/Kultur, though. Finally, it also gets four of the discrete emotions quite right, but still mingles joy/Freude and anger/Wut.

Interestingly, the bigger RA2 and RA3 models show more overlap between concrete, abstract, and especially emotion concepts. In RA2, one can see an approximate linear separability between animate and inanimate concrete concepts, but it mingles man/Mann and dog/Hund quite a bit. RA2 well separates abstract concepts clustering homeland/Heimat near peace/Frieden, sense/Sinn near courage/Mut, and information/Information apart. Like RA1, it still lacks a concept for culture, but unlike RA1, it mingles the basic emotions quite a bit. In this respect, RA3 seemingly mixes up all six emotion concepts.

To summarize, Study 2 established clear differences between the control “adult” model and the child models suggesting that especially emotion concepts get fuzzier with increasing vocabulary. The effect likely is due to the increasing number of different emotion terms used in RA2 and RA3 books. Theoretically, this could correspond to an increasing differentiation of emotion terms with increasing age. However, how and when infants, children, and adolescents develop emotion categories is not yet well-understood, and some authors argue that discrete categories are not learned at all (Hoemann et al., 2020).

To what extent the present exemplary data can be generalized to other emotion terms and book corpora (e.g., GLEC), and whether they correspond with the development of the emotion term vocabulary in children are key questions for future research. Whereas the neural and affective-cognitive processes underlying the codevelopment of language and emotion is still a badly underresearched area of psychology and the cognitive neurosciences (e.g., Sylvester et al., 2016, 2021a,b), a recent study suggests that the emotion vocabulary of children of age 4–11 doubles about every second year (Nook et al., 2017, 2020). According to this study, emotion representations develop from a unidimensional focus on valence to a bidimensional focus on both valence and arousal from age 6 to 25. Increasing the emotion vocabulary seems to mediate the development of emotion representations over and above other potential mediators and aids the expansion of emotion concepts from a “positive or negative” dichotomy in childhood to a multidimensional organization in adulthood. Study 2 provides an example of how DSMs could be used in such studies to generate testable quantitative predictions for this multidimensional organization.

To complement the qualitative analyses of Study 2, in Study 3, all DSMs were submitted to extensive quantitative tests (benchmarks) available for German.

## Study 3. Intelligence and Linguistic Tests (Semantic and Syntactic Knowledge)

As potential models of readers' AM, our DSMs should be able to perform a number of tasks used in intelligence or linguistic test batteries, such as analogical reasoning or correct verb conjugation. This can be examined with standard question and answer procedures discussed below. It should be noted that as far as we can tell all such NLP benchmark tests are based on and made for “adult” models and thus favor the control model (sdewac). What matters here is to see to what extent the performance of the child models changes with simulated reading age.

## Semantic Tests: Best Fit (Match), Does not Fit, And Opposite Problems (BF, DF, OP)

Solving analogies like “King is to man as X is to woman” is a first standard test of the quality of a DSM (best fit or best match problem; Mikolov et al., 2013). Another is the so-called does not fit (or odd item out) problem where one has to select the concept that does not fit to the other three, for example, France, Germany, Italy, and Africa. A final semantic test applied here concerns finding the correct opposite given an example pair, for example, old-young, night-X. Here, we used the 950 semantic question problems (540 best match, 110 does not fit, and 300 opposite questions) proposed by Mueller (2015, https://devmount.github.io/GermanWordEmbeddings/) to check the quality of his “german.model” (examples for English can be found at: https://github.com/nicholas-leonard/word2vec/blob/master/questions-words.txt). It was interesting to see whether the ability to solve these problems increased from RA1 to RA3 as the ability of children in the corresponding age groups clearly does. The performance of our four models in answering the overall 950 questions is given in Table 2.

**Table 2:** Semantic and morphosyntactic test performance.

	**RA1**	**RA2**	**RA3**	**sdewac**
BF	52, 10, 21	66, 15, 25	73, 11, 25	99, 58, 78
DF	66, 75, –	69, 84, –	71, 76, –	91, 82, –
OP	85, 7, 14	93, 24, 37	100, 20, 41	100, 36, 66
Mean correct matches % semantic	30.6	41	35.6	58.6
Morphosyntactic	68, 5	81, 12	88, 17	97, 61

*The 1st number refers to the hit rate or coverage, the 2nd to the % of correct matches, and, if present, the 3rd indicates whether the correct answer was among the top 10 semantic neighbors*.

As can be seen in Table 2, all four DSMs achieve a high accuracy in the DF test (75-84%), whereas the other two tests (BF and OP) pose problems for all models. The sdewac solves ~60% of analogies (BF) correctly, the three CL models only between 10 and 15%, whereas the correct answer is among the top 10 neighbors in 20–25% of the cases. In finding opposites, sdewac is correct in 36%, the CL models in only 7–24% with the correct answer being among the top 10 neighbors in 14–41% of the cases. With an average accuracy of 58.6% sdewac wins the semantic test competition, followed by RA2 (41%), RA3 (35.6%), and RA1 (30.6%). Thus descriptively, there is no clear progression from RA1 to RA3, but given the varying hit rates of the models—both across models and the three tests—and in the absence of inference statistics, this ranking is only exploratory or heuristic.

## Morphosyntactic Tests

Whereas, there are various standard semantic tests for DSMs, syntactic tests are less frequent in the literature. Mueller (2015) developed an extensive list of 10.000 morphosyntactic question problems for German, subdivided into 20 classes of 500 questions each, such as the building of plural (e.g., fear, fears, man, **men**), conjugation of verbs in present or past tense (e.g., go, goes, learn, **learns;** go, went, learn, **learned**), or degrees of adjectives (e.g., good, better, bad, **worse**). As shown in Table 2, with a hit rate of 97% reflecting the questions covered by the vocabulary of the model, the control model achieved an accuracy of 61.3% in these tests. The RA1 model achieved only 5% accuracy with a hit rate of 68%. RA2 produced 12% correct answers (hit rate = 81%), and RA3 17% (hit rate = 88%). Given that the control model using the same algorithm as our CL models performed not so badly, their poor performance regarding morphosyntactic questions may be due to the fact that the CL books do not contain sufficient training examples for the extensive questions developed by Mueller (2015).

In sum, compared with the control “adult” model, the three CL models still have a lot to learn. The observed difference between the RA1 model on the one hand, and the RA2 and RA3 models on the other, offers space for speculation. Thus, one could surmise that having read the ~200 books of RA1 with a resulting limited vocabulary of “only” ~50k words is a pretty good basis for developing concrete, abstract, and emotion concepts and also for solving “odd item out” puzzles (i.e., 75% correct answers to does not fit/DF questions), but not for correctly answering other semantic and morphosyntactic questions (~90% failures). Adding the knowledge compressed in 300 more CL books about doubles the vocabulary and the likelihood of correctly answering all kinds of semantic and morphosyntactic questions.

Lacking an empirical database providing corresponding results for human performance in these 950 semantic and 10.000 morphosyntactic test questions—something which could at least partly be done in future studies—in our final study, we examined how well our models fared in predicting human data from a series of rating experiments on word similarity and word valence.

## Study 4. Predicting Human Rating Data

Two standard crossvalidation tasks or benchmarks for the predictive validity of DSMs are human data on interword relatedness (“semantic similarity”) and word valence ratings (e.g., Turney and Littman, 2003; Baroni et al., 2014; Westbury et al., 2015). Whereas, alternative models have been reported to produce better fits to similarity ratings or also word association data than DSMs (e.g., Jacobs and Kinder, 2018b; De Deyne et al., 2019, 2021), their superior fit to valence rating data not only for single words (e.g., Hollis et al., 2017) but also for lines of poetry or whole text segments (e.g., Jacobs, 2018b; Jacobs and Kinder, 2019) remains a big challenge for any competitor model type. Also, their predictive power in simulating human word association ratings (not associations themselves) and especially in simulating both *behavioral and neural* affective semantics is even more challenging for other model types (e.g., Hofmann and Jacobs, 2014; Westbury et al., 2015; Hofmann et al., 2018; Roelke et al., 2018). It is thus the latter ability of DSMs that makes them excellent candidates for sentiment analyses of complex literary texts, for example, in the context of neurocognitive poetics studies (Jacobs, 2015, 2019; Jacobs et al., 2020).

Here, we wanted to examine how our different CL models performed in these two benchmark tasks. For the word-pair relatedness task, we correlated the cosine word vector similarities for all 350 word pairs from the most extensive German relatedness dataset known to us (Gurevych, 2005) for the three CL models and the control. This dataset was collected from adults.

For the valence rating task, we chose the “kidBAWL” dataset (Sylvester et al., 2016, 2021a,b) and correlated the computed *affective-aesthetic potential* (AAP) values for each word—following the procedure outlined in previous work (Jacobs, 2017, 2019)—with the children's rating data. The AAP is a potential, that is, a theoretical measure independent of reader responses, based on cosine similarities between the vectors of a given test word and those of a set of 120 predefined labels representing positive and negative affective-aesthetic concepts. The valence rating data were collected from children of age seven to 12.

**Figure 5:**
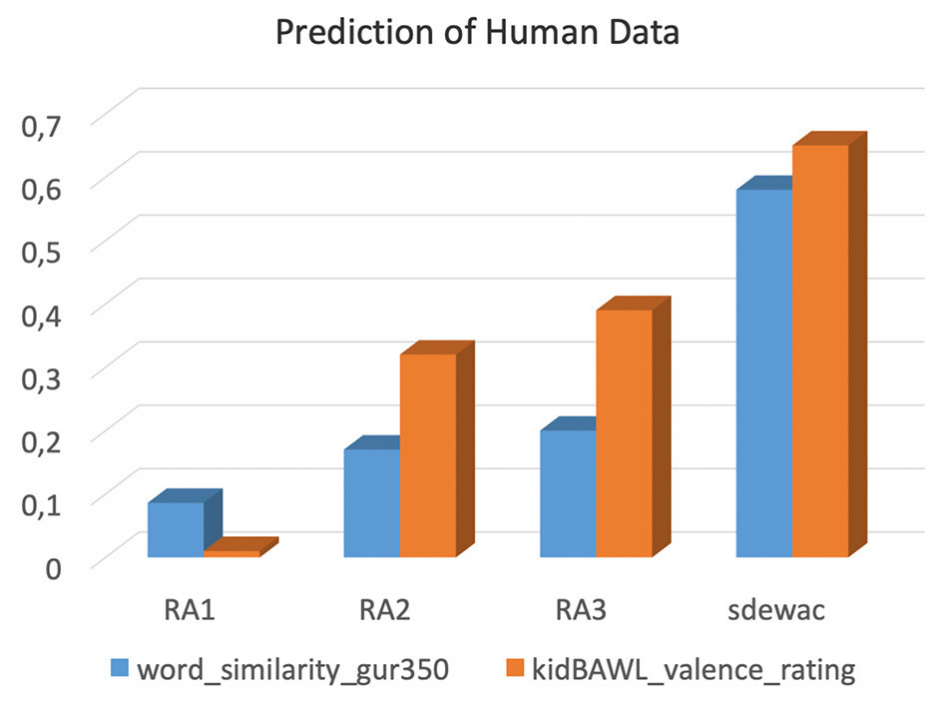
Performance of the four models (% accuracy) in predicting human rating data for a word similarity (blue) and valence decision task (orange).

Figure 5 summarizes the results of these two crossvalidation studies. The correlations for the word relatedness data were all significant, the best fitting “adult” model (sdewac) accounting for 60% of the variance in the response measure (quadratic fits: RA1. R^2^ = 0.091, *p* < 0.0002; RA2. R^2^ = 0.17, *p* < 0.0001; RA3. R^2^ = 0.23, p < 0.0001; sdewac. R^2^ = 0.6, *p* < 0.0001). As far as we can tell, this correlation of r >0.77 can challenge many an alternative unsupervised model, thus keeping DSM models of the present kind in the competition for this benchmark task. As could be expected, the child models achieve better fits with increasing simulated reading age, but RA3's performance, albeit being statistically significant, remains far below that of the adult control model.

The correlations for the word valence data also all were significant, the best fitting model (sdewac) accounting for almost 70% of the variance in the dependent variable (quadratic fits: RA1. R^2^ = 0.14, *p* < 0.002; RA2. R^2^ = 0.33, *p* < 0.0001; RA3. R^2^ = 0.4, *p* < 0.0001; sdewac. R^2^ = 0.67, *p* < 0.0001). Thus, prediction accuracy increased from RA1 to RA3, but sdewac outperformed all three CL models. This is not surprising since, on the one hand, adults usually perform better in any test than children, and on the other hand, because valence ratings in children are not solely based on the books they read, but also on multiple other sources of information, including their social interactions with adults (Sylvester et al., 2016, 2021a,b).

Overall, model fits were better for this dataset than for the previous one confirming the view that DSMs are viable predictive models for human affective semantics (e.g., Jacobs, 2019). Also, despite the blurred (discrete) emotion concept representations suggested by the TSNE method of Study 2, the RA3 model performs pretty well in this elementary binary affective decision task (positive vs. negative), which theoretically requires access to only two discrete emotions, joy and disgust (cf. Jacobs et al., 2016). Further combined computational and neuroscientific studies should look into this apparent discrepancy using other training corpora, model hyperparameters, or dimension reduction methods to shed light on the highly interesting relation between neural emotion concept representations and performance in the valence decision and (discrete) emotion recognition tasks.

## Discussion, Limitations, and Outlook

We computed DSMs based on a representative corpus of German children and youth literature as null models of the AM for readers of different reading ages. In Study 1, we used a measure of the overall semantic complexity of the three subcorpora (RA1, RA2, and RA3) and established RA3 as the most complex one suggesting that this model performs best in various semantic tasks used in intelligence or language tests, such as analogical reasoning. In three more studies, we then tested the quality of the models and evaluated their performance extensively in semantic and morphosyntactic tests, and also in predicting human data from word relatedness and valence ratings.

The extensive standard semantic and syntactic benchmark tests of Study 3 showed a remarkable performance of the three child models in the “does not fit” test (i.e., finding the odd item in sets of four), but great difficulties in the “best fit” and “opposite problems” and also in the morphosyntactic tasks. Given the decent performance of the “adult” control model, these data show that reader models that incorporate the vocabulary and semantic and syntactic knowledge of 200 to 500 children and youth books cannot compete with a control model that is based on 45 million sentences and possesses a vocabulary of ~1.5 million unique words (types). When applied to predict human rating data (Study 4), our models showed a remarkable performance, especially for word valence ratings indicating that knowledge of basic affective semantics is well-developed in the DSMs of the 500 CL books.

Overall, with increasing reading age performance became better in practically all tasks. Does this confirm the intuition that “the more books (of literary quality) one reads, the better one gets in intelligence or linguistic tests?” Given that the knowledge incorporated in DSMs likely represents only a tiny part of the cognitive and biosociocultural development children undergo from RA1 to RA3, our prudent answer is “well, it surely does not hurt.” At any rate, the approach taken in these studies reveals the potential and limits of DSMs for simulating human AM which we will discuss next.

## Modeling the Semantic Memory, Mental Lexicon, or Apperceptive Mass

Given the theme of this research topic, some more general considerations on psychological and computational terminology and models are in order. First, even almost 30 years after Elman's (1990, 2004) pioneering work on recurrent nets and his alternative view of the mental lexicon—seeing words as operators rather than as operands—there is no such thing as a standard conceptual, mathematical, computational, or other model of human *semantic memory* or the *mental lexicon* in particular. A scan through the multiple issues of journals such as “The Mental Lexicon,” “Psychological Review,” or others reveals a myriad of models of all types and colors (e.g., conceptual, mathematical, connectionist/deep neural nets, graph-theoretic, holographic, prototype vs. instance-based, count vs. predict DSMs, transformer/masked language models), and of methods to test them (for relevant reviews see, e.g., Jamieson et al., 2018; Linzen and Baroni, 2020; Kumar, 2021). That makes comparisons and benchmark competitions very hard. The standards for model evaluation proposed in earlier work (e.g., Hofmann and Jacobs, 2014) are yet far from being in practice, especially for computational models. Apart from a lack of standard measures of model complexity, falsifiability, descriptive, and explanatory adequacy, or vertical and horizontal generality (Jacobs and Grainger, 1994), even fundamental issues such as the “Turk problem” (Jones et al., 2015)—that is the “man in the machine” issue of using human behavioral data as a cognitive model's mental representation—or the “circularity problem” (e.g., Westbury, 2016; Hofmann et al., 2018)—for example, predicting human ratings with other human rating data incorporated in a model—remain unsolved. Moreover, the equally fundamental issue to what extent scientific models should find a balance between exploration, prediction, and explanation (Yarkoni and Westfall, 2017; Cichy and Kaiser, 2019) also remains open.

This being said, the question to what extent DSMs represent viable psychological models of human semantic memory, the mental lexicon or the AM is an ongoing one. In general, DSMs may be both too little (e.g., underestimating the contribution of speech, embodied experiences, etc.) and too much (e.g., often too big to be realistic). Still, DSMs can be considered as “serious contenders as psychological theories of semantic representation…” (Günther et al., 2019, p. 9)—and the AM in particular (Kintsch and Mangalath, 2011)—given that the representations they produce are at least partially grounded (e.g., Durda et al., 2009; Louwerse, 2011). Thus, DSMs are *representational* and not *process* models, although they have been considered as process models of the *acquisition* stage (Burgess, 2000)—albeit to a lesser degree than other neural nets (Huebner and Willits, 2018). They also can be used for process models, for example, by integrating them into broader theoretical frameworks, such as the instance theory of semantics (Jamieson et al., 2018), the construction-integration model (Kintsch and Mangalath, 2011), or the interactive activation framework (Hofmann et al., 2011; Hofmann and Jacobs, 2014). A recent model by Rotaru et al. (2018) presents a promising example for bringing together DSMs and spreading activation models, thus allowing to simulate performance in both automatic lexical processing (e.g., lexical and semantic decisions) and more deliberate processing (e.g., ratings).

In sum, as acquisition and representational null models allowing exploratory and predictive studies of the AM in the sense of Kintsch (1980), for example, for simulating children's semantic memory (Denhière and Lemaire, 2004), the present DSMs appear viable computational tools, but not as realistic online language processing models that explain, for example, reading behavior. Integrating them into broader frameworks such as spreading activation (Rotaru et al., 2018), or by concatenating them with novel transformer models (Alghanmi et al., 2020)—to overcome the limitations due to their static representation of word meaning—or with image embeddings (De Deyne et al., 2021)—to overcome their unimodality—bears a lot of promise for a multitude of applications in scientific studies of literature, research in education, or developmental science (cf. Kumar, 2021). Thus, as mentioned above, they could be used in neurocomputational studies of the emotional and linguistic development of children (e.g., Sylvester et al., 2021b), as quantitative predictors of the semantic complexity, literary quality, and readability or comprehensibility of texts, including school books (e.g., van Cranenburgh et al., 2019), or the aesthetic appreciation of poetry (Jacobs, 2018a,b).

## Data Availability Statement

The raw data supporting the conclusions of this article will be made available by the authors, without undue reservation.

## Author Contributions

AJ programmed the code. All authors conceived and wrote the manuscript together. All authors contributed to the article and approved the submitted version.

## Conflict of Interest

The authors declare that the research was conducted in the absence of any commercial or financial relationships that could be construed as a potential conflict of interest.

## Publisher's Note

All claims expressed in this article are solely those of the authors and do not necessarily represent those of their affiliated organizations, or those of the publisher, the editors and the reviewers. Any product that may be evaluated in this article, or claim that may be made by its manufacturer, is not guaranteed or endorsed by the publisher.

## Footnotes

^1^All models are available from the 1st author *via* email request.

^2^To obtain the final score for say two given books, one first divides them into N equal chunks of 1,000 sentences and then transforms these into vectors, as computed by the doc2vec model (Le and Mikolov, 2014). The vectors are then fed into a clustering algorithm (e.g., kmeans) to compute the cluster centers (centroids) and predict the cluster index for each book. A PCA is applied to the vectors to allow a 2d visualization. Finally, one computes the Euclidean distance between chunk c and chunk c+1 for all chunks of a given book and takes the mean of these distances as the final score. A greater Euclidian distance between neighboring chunks reflects a greater semantic difference between them, which translates into an overall higher semantic complexity for the entire book (cf. van Cranenburgh et al., 2019).

^3^The hyperparameters for our TSNE application were as follows: topn = 50, perp = 50, init = “pca,” learning_rate = 200.0, n_iter = 3,500, n_iter_without_progress = 300, random_state = 42.
